# Assessing the Impact of Neighborhood Socioeconomic Characteristics on COVID-19 Prevalence Across Seven States in the United States

**DOI:** 10.3389/fpubh.2020.571808

**Published:** 2020-09-22

**Authors:** Elham Hatef, Hsien-Yen Chang, Christopher Kitchen, Jonathan P. Weiner, Hadi Kharrazi

**Affiliations:** Department of Health Policy and Management, Center for Population Health Information Technology, Johns Hopkins Bloomberg School of Public Health, Baltimore, MD, United States

**Keywords:** coronavirus disease 2019, COVID-19, health disparities, social determinants of health, neighborhood characteristics

## Abstract

**Introduction:** The spread of Coronavirus Disease 2019 (COVID-19) across the United States has highlighted the long-standing nationwide health inequalities with socioeconomically challenged communities experiencing a higher burden of the disease. We assessed the impact of neighborhood socioeconomic characteristics on the COVID-19 prevalence across seven selected states (i.e., Arizona, Florida, Illinois, Maryland, North Carolina, South Carolina, and Virginia).

**Methods:** We obtained cumulative COVID-19 cases reported at the neighborhood aggregation level by Departments of Health in selected states on two dates (May 3rd, 2020, and May 30th, 2020) and assessed the correlation between the COVID-19 prevalence and neighborhood characteristics. We developed Area Deprivation Index (ADI), a composite measure to rank neighborhoods by their socioeconomic characteristics, using the 2018 US Census American Community Survey. The higher ADI rank represented more disadvantaged neighborhoods.

**Results:** After controlling for age, gender, and the square mileage of each community we identified Zip-codes with higher ADI (more disadvantaged neighborhoods) in Illinois and Maryland had higher COVID-19 prevalence comparing to zip-codes across the country and in the same state with lower ADI (less disadvantaged neighborhoods) using data on May 3rd. We detected the same pattern across all states except for Florida and Virginia using data on May 30th, 2020.

**Conclusion:** Our study provides evidence that not all Americans are at equal risk for COVID-19. Socioeconomic characteristics of communities appear to be associated with their COVID-19 susceptibility, at least among those study states with high rates of disease.

## Introduction

The spread of Coronavirus Disease 2019 (COVID-19) across the United States has highlighted the long-standing health inequalities (1, 2). There are substantial variations in the COVID-19 hospitalization and death rates; neighborhoods with the highest proportion of racial/ethnic minorities and the most persons living in poverty are experiencing higher rates of hospitalization and death. Such trends are present both nationally (3, 4) and in many small geographic areas hardest hit by the pandemic (5, 6).

To examine the disproportionate burden of COVID-19 across communities in the country it is necessary to assess the susceptibility of those communities to COVID-19 and the impact of their socioeconomic characteristics on the spread and severity of the disease. Such assessment will help to mount an adequate response strategy to better support community attempts to mitigate the ongoing spread of COVID-19 and the possible resurgence of the disease (1, 5).

We assessed the impact of neighborhood socioeconomic characteristics on COVID-19 prevalence in selected states; Arizona (AZ), Florida (FL), Illinois (IL), Maryland (MD), North Carolina (NC), South Carolina (SC), and Virginia (VA). These states provided a daily count of confirmed COVID-19 cases at a zip-code level enabling the assessment of the neighborhood socioeconomic characteristics at a more granular level, compared to county or state level reporting in other states. We used Area Deprivation Index (ADI) (7), a composite measure to rank neighborhoods by their socioeconomic characteristics in a jurisdiction of interest, because the social determinants of health are interconnected and impact outcomes in aggregate (8). Using the ADI composite measure helped to assess the combination of social conditions and how they would impact the COVID-19 risk across different communities.

## Methods

We used data on the number of cumulative confirmed COVID-19 cases at the zip-code level and percentage of the population tested for COVID-19 in the seven states on two selected dates, May 3rd, 2020 (when COVID-19 prevalence had an upward trend across the country) and May 30th, 2020 (when COVID-19 prevalence presented a downward trend in selected areas) (9–15). Zip-code level data was not available on May 3rd in North Carolina and Virginia.

We used the 2018 American Community Survey (ACS), an annual nationwide profile conducted by the US Census Bureau (16), to construct ADI raw scores for all zip codes in the country. We then sorted and ranked the scores for each community (7). We developed ADI state ranks as deciles and national ranks as percentiles comparing each zip-code to others in the same state and across the country. The higher ADI rank represented more disadvantaged neighborhoods. We used U.S. Census data to identify estimates for population size, age, gender, and race distribution in the study states (17).

We identified variations in reporting confirmed cases across the states. For instance, some states provided categorical values wherever cases in a neighborhood fell below a certain threshold. To harmonize these variations, we assigned a mean value in each category to the present number of COVID-19 cases (see Supplementary Table for more details).

We calculated COVID-19 prevalence using the zip-code resident population as the denominator. We performed descriptive analyses and assessed correlations between COVID-19 prevalence with ADI (national and state ranks to control for state-specific characteristics) and race. We provided unadjusted correlation coefficients and adjusted ones controlling for age, gender, and the square mileage of each zip-code community to address the impact of population density on COVID-19 spread. Additionally, we evaluated the correlation between a 40-day (April 20th to May 30th, 2020) change in COVID-19 prevalence and ADI. Moreover, when data were available we assessed the correlation between the percentage of the population tested for COVID-19 in a zip-code and ADI national and state ranks.

We did not obtain institutional review board approval due to the use of publicly available, de-identified data, per usual institutional policy.

## Results

Table 1 presents an overview of population characteristics in selected states. IL and MD reported the highest COVID-19 prevalence on May 3rd (503.79 and 436.81 per 100,000 population, respectively) and May 30th (949.03 and 872.99 per 100,000 population, respectively). Both states had the highest number of the state population tested on May 3rd (2.63% and 2.26%, respectively) and May 30th (7.09% and 4.99%, respectively).

**Table 1:** An overview of population characteristics across seven states.

	**Arizona**	**Florida**	**Illinois**	**Maryland**	**North Carolina**	**South Carolina**	**Virginia**
**COVID-19 prevalence (per 100,000 population)**							
May 3rd, 2020	122.53	171.79	503.79	436.81	112.97	128.69	228.36
May 30th, 2020	273.89	261.49	949.03	872.99	272.58	230.37	522.60
**COVID-19 test availability (% population tested)**							
May 3rd, 2020	1.17	2.07	2.63	2.26	1.40	1.25	1.31
May 30th, 2020	3.09	4.75	7.09	4.99	3.97	3.89	3.69
**ADI national rank^a^**							
Mean ± 95% CI	59.1 ± 32.2	51.2 ± 28.9	47.0 ± 25.7	26.6 ± 25.3	60.4 ± 26.0	68.8 ± 25.4	45.6 ± 29.9
**Race**							
% of whites	54%	53%	61%	50%	63%	64%	62%
**ADI and race**							
**ADI national rank^b^** **(Mean** **±** **95% CI**)							
White majority zip-codes (white ≥75%)	46.65 ± 29.29	43.16 ± 27.28	45.29 ± 23.72	24.74 ± 24.00	51.67 ± 24.90	52.13 ± 27.16	44.64 ± 29.54
Racial minority zip-codes (white <75%)	76.32 ± 23.80	67.20 ± 24.64	56.94 ± 33.13	29.43 ± 27.01	71.54 ± 22.95	77.36 ± 18.07	47.27 ± 30.42
**ADI state rank^c^** **(Mean** **±** **95% CI)**							
White majority zip-codes (white ≥75%)	4.32 ± 2.42	4.70 ± 2.70	5.33 ± 2.72	5.31 ± 2.83	4.46 ± 2.55	3.63 ± 2.37	5.41 ± 2.83
Racial minority zip-codes (white <75%)	6.99 ± 2.23	7.10 ± 2.48	6.45 ± 3.50	5.80 ± 2.94	6.82 ± 2.73	6.38 ± 2.55	5.65 ± 2.94

a *ADI state rank has a mean of 5.5 and 95% CI of 2.9 for all states, due to the method for calculating the rank. Higher the rank, the greater the disadvantage*.

b *P-values comparing zip-codes with and without white majority were <0.0001 except for Maryland (P = 0.0537) and Virginia (P = 0.2051)*.

c *P-values comparing zip-codes with and without white majority were <0.0001 except for Maryland (P = 0.0769) and Virginia (P = 0.2376)*.

*ADI, Area Deprivation Index; CI, Confidence Interval*.

On May 3rd when the number of tested individuals and COVID-19 prevalence was relatively low across the selected states we detected positive and statistically significant correlations between COVID-19 prevalence and both national and state-ranked ADI in IL, MD, and SC without adjustment and IL and MD when adjustments for underlying demographics were made. Therefore, zip-codes with higher ADI (more disadvantaged neighborhoods) in those states had higher COVID-19 prevalence compared to zip-codes across the country and in the same state with lower ADI (less disadvantaged neighborhoods). On May 30th when the number of tested individuals and COVID-19 prevalence increased across the selected states we detected positive correlations between COVID-19 prevalence and both national and state-ranked ADI in all states except for VA without adjustment and when adjustments for underlying demographics were made. Therefore, more disadvantaged neighborhoods in those states had higher COVID-19 prevalence compared to less disadvantaged neighborhoods (Table 2).

**Table 2:** Correlation between COVID-19 prevalence and socioeconomic status on zip-code level across seven states.

	**Arizona**	**Florida**	**Illinois**	**Maryland**	**North Carolina**	**South Carolina**	**Virginia**
**May 3rd 2020^a^**							
**Correlation without adjustment^b^**							
ADI national rank	0.05 *P* = 0.3473	−0.05 *P* = 0.1236	0.09 *P* < 0.05	0.14 *P* < 0.05	NA	0.13 *P* < 0.05	NA
ADI state rank	0.05 *P* = 0.3237	−0.05 *P* = 0.1285	0.08 *P* < 0.05	0.14 *P* < 0.05	NA	0.14 *P* < 0.05	NA
Race (whites)	−0.40 *P* < 0.0001	−0.27 *P* < 0.0001	0.03 *P* = 0.249	−0.11 *P* < 0.05	NA	−0.28 *P* < 0.0001	NA
**Correlation with adjustment^c^**							
ADI national rank	−0.02 *P* = 0.7448	−0.07 *P* < 0.05	0.19 *P* < 0.0001	0.14 *P* < 0.05	NA	0.04 *P* = 0.4422	NA
ADI state rank	−0.02 *P* = 0.7685	−0.07 *P* < 0.05	0.19 *P* < 0.0001	0.14 *P* < 0.05	NA	0.05 *P* = 0.3125	NA
Race (whites)	−0.25 *P* < 0.0001	−0.19 *P* < 0.0001	0.15 *P* < 0.0001	−0.14 *P* < 0.05	NA	−0.21 *P* < 0.0001	NA
**May 30th 2020**							
**Correlation without adjustment^b^**							
ADI national rank	0.218 *P* < 0.0001	0.069 *P* = 0.035	0.026 *P* = 0.3309	0.160 *P* = 0.0006	0.324 *P* < 0.0001	0.241 *P* < 0.0001	−0.219 *P* < 0.0001
ADI state rank	0.220 *P* < 0.0001	0.070 *P* = 0.032	0.024 *P* = 0.3760	0.158 *P* = 0.0007	0.326 *P* < 0.0001	0.247 *P* < 0.0001	−0.219 *P* < 0.0001
Race (whites)	−0.452 *P* < 0.0001	−0.321 *P* < 0.0001	−0.158 *P* < 0.0001	−0.253 *P* < 0.0001	−0.450 *P* < 0.0001	−0.363 *P* < 0.0001	−0.369 *P* < 0.0001
**Correlation with adjustment^c^**							
ADI national rank	0.152 *P* = 0.0059	0.041 *P* = 0.2137	0.100 *P* = 0.0002	0.134 *P* = 0.0045	0.254 *P* < 0.0001	0.157 *P* = 0.0021	−0.178 *P* < 0.0001
ADI state rank	0.150 *P* = 0.0066	0.041 *P* = 0.2104	0.099 *P* = 0.0002	0.134 *P* = 0.0046	0.256 *P* < 0.0001	0.164 *P* = 0.0012	−0.177 *P* < 0.0001
Race (whites)	−0.224 *P* < 0.0001	−0.231*P* < 0.0001	−0.047 *P* = 0.0784	−0.225*P* < 0.0001	−0.358 *P* < 0.0001	−0.307*P* < 0.0001	−0.318 *P* < 0.0001

a *Zip-code level data were not available on May 3rd for North Carolina and Virginia*.

b *Spearman correlation coefficient and p-value*.

c *Correlation adjusted for age, gender, and the square mileage of each zip-code community*.

*ADI, Area Deprivation Index (The higher the Index, the greater the disadvantages)*.

**Figure 1:**
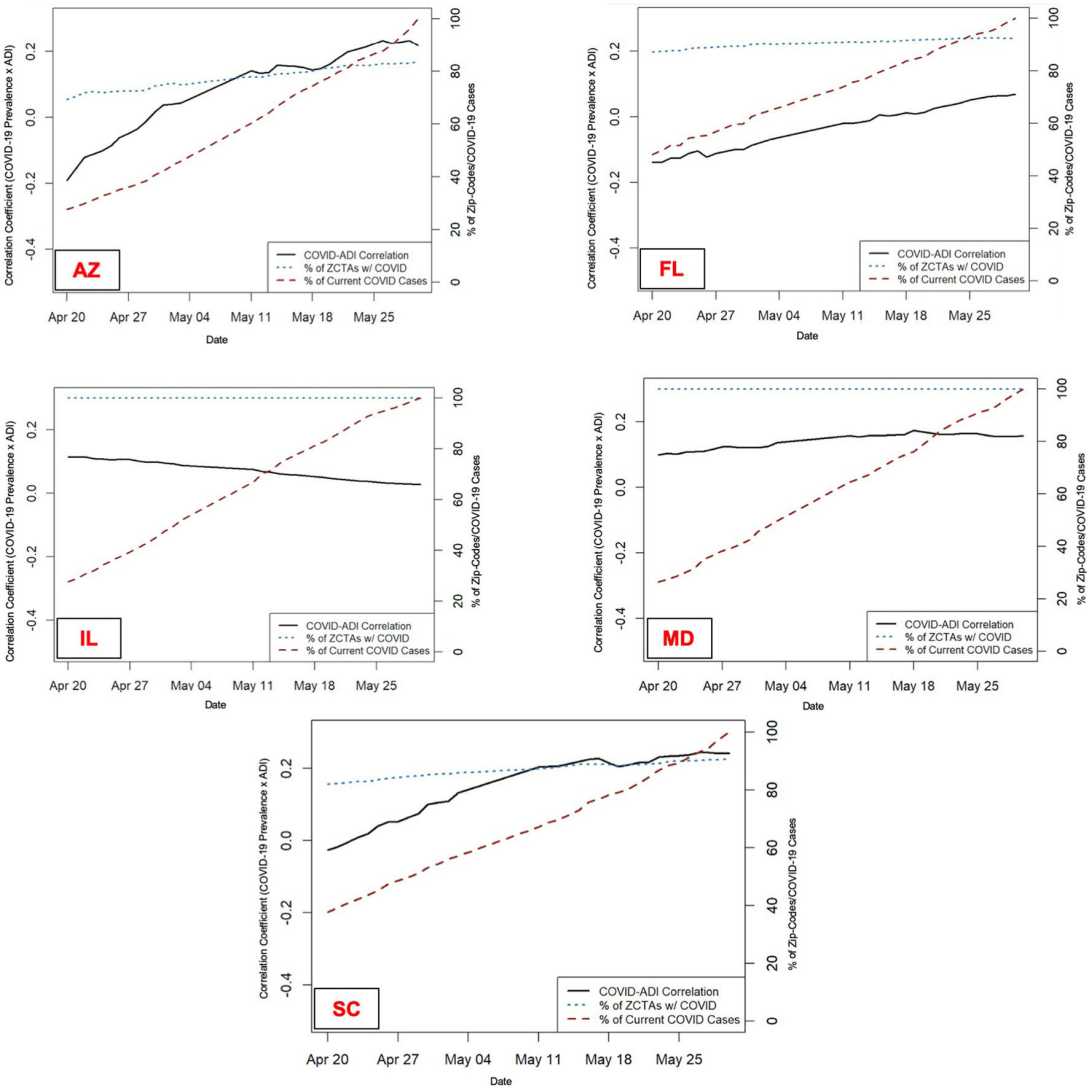
Change in the COVID-19 Prevalence and Correlation with ADI Over Time–Percentage of zip-codes with any COVID-19 cases (blue dotted line), COVID-19 prevalence (red dotted line), and the correlation between COVID-19 prevalence and ADI (black solid line) from April 20th to May 30th, 2020 across five selected states. AZ, Arizona; FL, Florida; IL, Illinois; MD, Maryland; SC, South Carolina. Zip-code level data were not available for North Carolina and Virginia during this period to perform longitudinal analysis.

Figure 1 presents the percentage of zip-codes with any COVID-19 cases (blue dotted line), COVID-19 prevalence (red dotted line), and the correlation between COVID-19 prevalence and ADI (black solid line) from April 20th to May 30th. The figure reveals the increasing correlation between COVID-19 prevalence and ADI in AZ, FL, and SC over time as COVID-19 spread across communities.

In terms of race, on May 3rd COVID-19 prevalence was negatively correlated with the proportion of the community being white in all states, except for IL without adjustment and when adjustments for underlying demographics were made. On May 30th COVID-19 prevalence was negatively correlated with the proportion of the community being white in all states without and with adjustments for underlying demographics (Table 2).

Assessing the correlation between the percentage of the population tested for COVID-19 in a zip-code and ADI national and state ranks revealed positive correlations in IL without adjustment and when adjustments for underlying demographics were made. Therefore, zip-codes with higher ADI (more disadvantaged neighborhoods) in IL had a higher percentage of their population tested for COVID-19 compared to zip-codes across the country and in IL with lower ADI (less disadvantaged neighborhoods). The correlation coefficients were negative in VA without adjustment and when adjustments for underlying demographics were made. Thus, more disadvantaged neighborhoods had a lower percentage of their population being tested for COVID-19 compared to less disadvantaged neighborhoods across the country and in VA (Table 3).

**Table 3:** Correlation between COVID-19 test availability and socioeconomic status on zip-code level in two states.

	**Illinois**	**Virginia**
**May 3rd, 2020^a^**		
**Correlation without adjustment^b^**		
ADI national rank	0.101 *P* = 0.0305	NA
ADI state rank	0.118 *P* = 0.0115	NA
**Correlation with adjustment^c^**		
ADI national rank	0.044 *P* = 0.3556	NA
ADI state rank	0.068 *P* = 0.1517	NA
**May 30th, 2020**		
**Correlation without adjustment^b^**		
ADI national rank	0.183 *P* < 0.0001	−0.104 *P* = 0.0036
ADI state rank	0.195 *P* < 0.0001	−0.103 *P* = 0.0042
**Correlation with adjustment^c^**		
ADI national rank	0.099 *P* = 0.0239	−0.118 *P* = 0.0010
ADI state rank	0.113 *P* = 0.0099	−0.118 *P* = 0.0011

a *Zip-code level data were not available on May 3rd for Virginia*.

b *Spearman correlation coefficient and p-value*.

c *Correlation adjusted for age, gender, and the square mileage of each zip-code community*.

*ADI, Area Deprivation Index (The higher the Index, the greater the disadvantages)*.

## Discussion

The positive and statistically significant correlation between COVID-19 prevalence and ADI in IL and MD before and after adjustment on May 3rd, 2020 confirms the higher burden of the disease in disadvantaged communities. Both states had higher proportions of their population being tested and more confirmed cases by May 3rd, 2020. In other states with the fewer number of tested individuals and lower COVID-19 prevalence we detected negligible correlation, the correlation was not statistically significant (e.g., AZ and FL before adjustment and SC after adjustment), and/ or reversed after taking into account age, gender, and the square mileage of each zip-code (AZ after adjustment). Using May 30th data when the number of tested individuals and COVID-19 prevalence increased across the selected states the correlation between COVID-19 prevalence and ADI was positive in all states except for VA. The negative correlation in VA could be attributed to the lower number of tested individuals in disadvantaged neighborhoods (Table 3). Therefore, the magnitude of the disease was not properly detected in VA disadvantaged communities.

These findings confirm the hypothesis that low levels of COVID-19 testing, especially among communities with socioeconomic challenges, might mask the higher burden of the disease in those communities. With an increase in the number and proportion of tested individuals, a higher burden of the disease among disadvantaged communities might gradually be revealed. The longitudinal assessment of the correlation also supported this hypothesis; the relative increase in COVID-19 prevalence in AZ, FL, and SC in the 40-day interval resulted in a stronger correlation with ADI. The same trend was detected in IL and MD with a relative increase in COVID-19 prevalence at an earlier time interval (not shown in Figure 1).

Our results were comparable with Wadhera et al. (5) findings. They identified higher COVID-19 burden (hospitalization and death) among residents of the Bronx, who also had lower income and a higher number of tested individuals comparing to other boroughs of New York City. Communities comprised mainly of racial minorities and those economically challenged may be more susceptible to severe forms of COVID-19 due to a higher prevalence of underlying conditions such as diabetes, hypertension, and cardiovascular disease (18). Such conditions have been associated with COVID-19 poor outcomes (19).

Data on race and ethnicity are incomplete across the US and only available through the efforts by local governments (20). Using the available data on May 3rd, 2020 we detected a higher correlation between COVID-19 prevalence and racial minorities in all states except for IL. In IL, COVID-19 prevalence was higher in whites comparing to racial minorities. The statistically significant difference in mean ADI ranks between the white majority and racial minority neighborhoods was lowest in IL compared to AZ, FL, and SC (Table 1). In other words, in IL the socioeconomic status of predominantly white neighborhoods was similar to communities comprised mainly of racial minorities. This finding might support the hypothesis that the higher COVID-19 prevalence among racial minorities is mostly due to their socioeconomic disadvantage rather than any race-linked clinical factors. Using May 30th data when the number of tested individuals and COVID-19 prevalence increased across selected states we detected a higher correlation between COVID-19 prevalence and racial minorities in all states.

This study has limitations, including a limited data set on a population level, a lack of data on socio-economic characteristics of COVID-19 patients, and variability in methods of reporting COVID-19 cases and data quality across selected states. We acknowledge that COVID-19 testing and prevalence are inter-connected and higher testing in specific communities might result in higher disease. Lack of adequate testing might lead to spurious results in the assessment of the correlation between COVID-19 prevalence and ADI.

Despite limitations, our study confirms that not all Americans are equally at risk for COVID-19. Socioeconomic characteristics of communities are likely to determine susceptibility to COVID-19 (1). Communities comprised mainly of racial minorities and economically challenged households are more likely to be exposed to COVID-19 due to their overrepresentation in the low-wage, essential work at the front lines such as in the healthcare system (20–22). For instance, low-wage healthcare workers often have multiple jobs at clinics, hospitals, and nursing homes which results in a dramatic increase in their COVID-19 risk (20). Moreover, social distancing is more challenging in socioeconomically disadvantaged neighborhoods with high housing density and overcrowding (20). Our findings highlight the vital role of world-class data and analytics to support disease surveillance and public health decision-making. The current outbreak of COVID-19 reminds us of the urgency of the modernization of the public health data enterprise to protect and promote the public's health (23). Policymakers need to recognize the variation in risks across different communities to mount an adequate response strategy. Strategies to protect the most vulnerable neighborhoods will require urgent measures to better assess and take into account their socio-economic challenges.

## Data Availability Statement

Publicly available datasets were analyzed in this study. This data can be found here: 1. Arizona Department of Health. Highlighted Infectious Diseases for Arizona. https://www.azdhs.gov/preparedness/epidemiology-disease-control/infectious-disease-epidemiology/index.php#novel-coronavirus-home. 2. Florida Department of Health, Division of Disease Control and Health Protection. Florida's COVID-19 Data and Surveillance Dashboard. https://experience.arcgis.com/experience/96dd742462124fa0b38ddedb9b25e429/. 3. Illinois Department of Public Health. Coronavirus Disease 2019 (COVID-19). http://www.dph.illinois.gov/topics-services/diseases-and-conditions/diseases-a-z-list/coronavirus. 4. Maryland Department of Health. Coronavirus Disease 2019 (COVID-19) Outbreak. https://coronavirus.maryland.gov/. 5. North Carolina Department of Health. NCDHHS' COVID-19 Response. https://covid19.ncdhhs.gov. 6. South Carolina Department of Health and Environmental Control. SC Testing Data and Projections (COVID-19). https://scdhec.gov/health/infectious-diseases/viruses/coronavirus-disease-2019-covid-19/monitoring-testing-covid-19. 7. Virginia Department of Health. COVID-19 in Virginia. https://www.vdh.virginia.gov/coronavirus/.

## Ethics Statement

Ethical review and approval was not required for the study on human participants in accordance with the local legislation and institutional requirements. Written informed consent for participation was not required for this study in accordance with the national legislation and the institutional requirements.

## Author Contributions

All authors contributed significantly to the project and writing of the manuscript. All authors reviewed the final paper and provided comments as deemed necessary. EH supervised the development of the analysis plan, reviewed and interpreted the results, and led writing this paper. H-YC and CK performed the data analysis. JW contributed to setting the overall scope and goal of the project as well as finalizing the manuscript. HK designed the overall scope and goals of the study and supervised the day-to-day operations of the project.

## Conflict of Interest

The authors declare that the research was conducted in the absence of any commercial or financial relationships that could be construed as a potential conflict of interest.
