# Supplementary material for: Assessing the Impact of Neighborhood Socioeconomic Characteristics on COVID-19 Prevalence Across Seven States in the United States
Source: Front Public Health. 2020 Sep 22;8:571808. doi: 10.3389/fpubh.2020.571808 (PMC7536340; doi:10.3389/fpubh.2020.571808)
Supplement: Supplementary file 1 [file Table_1.DOCX]

| **Addressing Variation in the Reporting Methods for Confirmed COVID-19 Cases Across Selected States.** | | | | |
| --- | --- | --- | --- | --- |
| **Value Reported by State Health Department** | **No. of Zip-Codes** | | **Revised Number** | **Comments** |
|  | **May 3^rd^, 2020** | **May 30^th^, 2020** |  |  |
| **Arizona** | | | | |
| 1-5 COVID-19 Cases | 59 | 66 | 3 | Provided 0 for zip-codes with no cases and categorical values for zip-codes with ≤ 10 cases |
| 6-10 COVID-19 Cases | 28 | 21 | 8 |  |
| Number of Cases Suppressed | 38 | 38 | Missing | Tribal approval is pending |
| Not included in state report / Had valid ADI rank | 27 | 27 | Missing |  |
| Included in state report / No valid ADI rank^a^ | 8 | 8 | Missing |  |
| A mismatch between state report and ADI rank | 3 | 3 | Missing |  |
| **Florida**^b^ | | | |  |
| <5 COVID-19 Cases | 153 | 133 | 2.5 | Provided 0 for zip-codes with no cases and actual case number on a zip-code level only in counties with ≥ 5 cases |
| Number of Cases Suppressed | 18 | 9 | 0 |  |
| Not included in state report / Had valid ADI rank | 40 | 40 | Missing |  |
| Included in state report / No valid ADI rank^a^ | 20 | 20 | Missing |  |
| **Illinois** | | | | |
| Not included in state report / Had valid ADI rank | 931 | 858 | 2.5 | Excluded zip-codes with <6 cases |
| Included in state report / No valid ADI rank^a^ | 2 | 3 | Missing |  |
| A mismatch between state report and ADI rank | 3 | 2 | Missing |  |
| **Maryland** | | | | |
| Not included in state report / Had valid ADI rank | 237 | 177 | 3.5 | Excluded zip-codes with <8 cases |
| **North Carolina**^c^ | | | | |
| Not included in state report / Had valid ADI rank | NA | 116 | 0 |  |
| Included in state report / No valid ADI rank^a^ | NA | 57 | Missing |  |
| **South Carolina** | | | | |
| Not included in state report / Had valid ADI rank | 30 | 30 | Missing |  |
| Included in state report / No valid ADI rank^a^ | 2 | 2 | Missing |  |
| **Virginia**^c^ | | | | |
| Number of Cases Suppressed | NA | 236 | 2.5 |  |
| Not included in state report / Had valid ADI rank | NA | 9 | Missing |  |
| A mismatch between state report and ADI rank | NA | 1 | Missing |  |
| ^a^ The algorithm for calculating ADI dropped any zip-code with fewer than 100 persons, fewer than 30 housing units, or greater than 33% of the population living in group quarters.  ^b^ Florida provided multiple records per zip-code; the counts reported in the table are at the record level instead of zip-code. We aggregated multiple records per zip-code and reported one record per zip-code.  ^c^ Zip-code level data was not available on May 3^rd^ for North Carolina and Virginia.  ADI: Area Deprivation Index, COVID-19: Coronavirus Disease 2019 | | | | |
